# Lincoln’s Religion

**Published:** 1889-08

**Authors:** 


### LINCOLN'S RELIGION.

The August number of the *Century* will contain a chapter on "Lincoln and the Churches" in the Lincoln History, by Messrs. Hay and Nicolay, from which the following is an extract from advance sheets :

He was a man of profound and intense religious feeling. We have only to look at his authentic public and private utterances to see how deep and strong in all the latter part of his life was the current of his religious thought and emotion. The pressure of the tremendous problems by which he was surrounded, the awful moral significance of the conflict in which he was the chief combatant ; the overwhelming sense of personal responsibility, which never left him for an hour—all contributed to produce, in a temperament naturally serious and predisposed to a spiritual view of life and conduct, a sense of reverent acceptance of the guidance of a Superior Power. From that morning when, standing amid the falling snowflakes on the railway car at Springfield, he asked the prayers of his neighbors in those touching phrases whose echo rose that night in invocations from thousands of family altars, to that memorable hour when on the steps of the Capitol he humbled himself before his Creator in the sublime words of the second inaugural, there is not an expression known to have come from his lips or his pen, but proves that he held himself answerable in every act of his career to a more august tribunal than any on earth. The fact that he was not a communicant of any church, and that he was singularly reserved in regard to his personal religious life, gives only the greater force to these striking proofs of his profound reverence and faith.

